# 

**Published:** 1888-10

**Authors:** 


The Buffalo Medical and Surgical Association.—Programme of winter meetings, November, 1888, to April, 1889. Meetings held on the first Tuesday in each month. November 6th, W. A. Wheeler, M. D., "Certain Diseases of the Testicle;" H. R. Hopkins, M. D., "Basedow's Disease." December 4th, F. H. Potter, M. D., "Treatment of Acute Coryza;" Roswell Park, M. D., "Radical Cure of Hernia." January 1st, Ernest Wende, M. D., "Parasitic Skin Diseases;" W. C. Phelps, M. D., "Surgery of the Knee-Joint." February 5th, "Syphilis: Its Later Manifestations and its Curability;" Discussion by Dr. Charles G. Stockton, "Medical, including Nervous;" Dr. Roswell Park, "Surgical;" Dr. William H. Heath, "Genito-Urinary;" Dr. Elmer G. Starr, "Eye and Ear;" Dr. F. H. Potter, "Throat;" Dr. Ernest Wende, "Skin;" Dr. M. D. Mann, "Heredity." March 5th, H. E. Hayd, M. D., "Impotency: Its Causes and Treatment;" Lucien Howe, M. D., "Bacteria in Conjunctivitis." April 2d, W. H. Heath, M. D., "Frequent

Micturition and its Treatment;" J. Falk, M. D., "The Toxic Effect of Alcohol and Tobacco on the Eyes." Officers for 1888–9: *President*, Dr. P. W. Van Peyma; *Vice-President*, Dr. A. A. Hubbell; *Secretary*, Dr. W. H. Bergtold; *Treasurer*, Dr. F. E. L. Brecht; *Librarian*, Dr. Lucien Howe.

---

THE SEVENTH ANNUAL ANNOUNCEMENT of the New York Polyclinic and Hospital, a clinical school for graduates in medicine and surgery, has been received. The class for the session of 1887–8 numbered 337, an increase of thirty-six over the preceding year. The changes in the Faculty are the appointments of Dr. Henry N. Heinemann, Professor of General Medicine, and Dr. Charles Stedman Bull, Professor of Ophthalmology. The New York Polyclinic Hospital will be opened in October. The preliminary term begins September 17th, and the regular term on September 24th.

---

THE SOUTHERN SURGICAL AND GYNECOLOGICAL ASSOCIATION has postponed its annual meeting until the first Tuesday in December next. This action became necessary on account of the quarantine against yellow fever. The meeting will be held in Birmingham, Ala.

### BOOKS AND PAMPHLETS RECEIVED.

Disinfection and Disinfectants, etc. Published by the Committee on Disinfectants of the American Public Health Association, Concord, N. H. 1888.

New York State Board of Health. Eighth Annual Report. 1888.

Same. Monthly Bulletin. August, 1888.

University of Nebraska. Second Annual Report of the Patho-Biological Laboratory. The Swine Plague. By Dr. Frank S. Billings, Director, Lincoln, Neb. 1888.

Transactions of the Medical Association of the State of Missouri. 1888.

Annual Report of the Commissioner of Pensions. 1888.

Weekly Abstract of Sanitary Reports. U. S. Marine Hospital Service. Abstracts 35, 36, 37, 38 and 39. 1888.

Merck's Bulletin. August, 1888.

Suicide and Legislation. By Clerk Bell, Esq., President Medico-Legal Society, State of New York. Author's reprint. 1888.

The Prevention and Cure of Perineal Lacerations. By Dr. Thomas Opie, Baltimore, Md. Author's reprint. 1887.

Electricity vs. Tait, etc. By Dr. George F. Hulbert, St. Louis. Author's reprint. 1888.

Two Cases of Removal of Uterine Myoma, etc. By Dr. Mary A. Dixon Jones, Brooklyn. Author's reprint. 1888.

Removal of the Uterine Appendages. Nine consecutive cases. Same. 1886.

A Case of Poisoning by Sulphate of Atropia; Recovery. By Dr. Llewellyn Eliot, Washington. Author's reprint, 1888.

Ueber Tuberkel-Knoten des Cerebellums. Inaugural Dissertation. Der Friederich Wilhelms Universität. By Dr. William Christopher Krauss, of Attica, N. Y. Berlin. 1888.

Michigan State Board of Health Bulletin. August, 1888.

Subcutaneous Division of Urethral Stricture. By Dr. Claudius H. Mastin, Mobile, Ala. Author's reprint. 1886.

The American Hip-Splint. By Dr. A. B. Judson, New York. Author's reprint. 1888.

Tennessee State Board of Health Bulletin. August, 1888.

Vascular Growths of the Female Meatus Urinarius. By Dr. Augustus P. Clarke, Cambridge. Author's reprint. 1888.

---

### COLLEGE ANNOUNCEMENTS.

Starling Medical College, Columbus, Ohio. (Forty-second.) 1888.

The New York Polyclinic and Hospital. (Seventh.) 1888.

The Bennett Medical College, Chicago. (Eclectic. Twenty-first.) 1888.

